# Supplementary material for: Implementation strategies and economic considerations for point-of-care ultrasound in Low- and Middle-Income Countries: A scoping review
Source: PLOS Glob Public Health. 2026 Feb 9;6(2):e0005852. doi: 10.1371/journal.pgph.0005852 (PMC12885260; doi:10.1371/journal.pgph.0005852)
Supplement: S1 Text — (DOCX) [file pgph.0005852.s002.docx]

**S1 Text: Search Strategy**

The search strategy is based on three terms: point of care ultrasound, implementation strategies and developing countries. A search strategy for PubMed/Medline is presented below. This strategy was adapted for the additional databases included.

Version 1

((Afghanistan OR Algeria OR Angola OR Bangladesh OR Benin OR Bhutan OR

Bolivia OR "Burkina Faso" OR Burundi OR "Cabo Verde" OR Cambodia OR

Cameroon OR "Central African Republic" OR Chad OR Comoros OR "Democratic

Republic of Congo" OR "Republic of Congo" OR "Cote d’Ivoire" OR Djibouti OR

"Arab Republic of Egypt" OR Egypt OR Eritrea OR Eswatini OR Ethiopia OR "The

Gambia" OR Ghana OR Guinea OR "Guinea-Bissau" OR Haiti OR Honduras OR

India OR "Islamic Republic of Iran" OR Iran OR Jordan OR Kenya OR Kiribati OR

"Democratic People’s Republic of Korea" OR Korea OR "Kyrgyz Republic" OR

"Lao PDR" OR Lebanon OR Lesotho OR Liberia OR Madagascar OR Malawi OR

Mali OR Mauritania OR Micronesia OR Mongolia OR Morocco OR Mozambique

OR Myanmar OR Nepal OR Nicaragua OR Niger OR Nigeria OR Pakistan OR

"Papua New Guinea" OR Philippines OR Rwanda OR Samoa OR "Sao Tome and

Principe" OR Senegal OR "Sierra Leone" OR Solomon Islands OR Somalia OR

"South Sudan" OR "Sri Lanka" OR Sudan OR "Syrian Arab Republic" OR Syria

OR Tajikistan OR Tanzania OR "Timor-Leste" OR Togo OR Tunisia OR Uganda

OR Ukraine OR Uzbekistan OR Vanuatu OR "Viet Nam" OR Vietnam OR

"Republic of Yemen" OR Yemen OR Zambia OR Zimbabwe) OR ("Low-resource

countries" OR "Low resource countries" OR "Developing countries" OR "Low

and Middle income countries" OR LMIC OR "Low-resource settings" OR "Low

resource settings" OR "Lower middle income countries")) AND

("implementation strategy" OR "Implementation strategies" OR "Setting up" OR

"Establishing strategies" OR "Establishing" OR "Cost benefit" OR "Cost

effectiveness") AND ("point of care ultrasound" OR "Point-of-care ultrasound"

OR "Point of care ultrasonography" OR "Point-of-care ultrasonography" OR

"Bedside ultrasound" OR "Bedside ultrasonography" OR "ultrasound" OR

"Ultrasonography" OR "Sonography")
