# Supplementary material for: Implementation strategies and economic considerations for point-of-care ultrasound in Low- and Middle-Income Countries: A scoping review
Source: PLOS Glob Public Health. 2026 Feb 9;6(2):e0005852. doi: 10.1371/journal.pgph.0005852 (PMC12885260; doi:10.1371/journal.pgph.0005852)
Supplement: S2 Text — (DOCX) [file pgph.0005852.s003.docx]

**S2 Text: Critical appraisal tool**

Critical appraisal tools for quasi-experimental studies and economic evaluations adapted from JBI.

Critical appraisal tool for quasi-experimental studies:

| Item | Yes | Partially | No | Unclear | N/A |
| --- | --- | --- | --- | --- | --- |
| Is it clear in the study what is the ‘cause’ and what is the ‘effect’ (i.e. there is no confusion about which variable comes first)? | ☐ | ☐ | ☐ | ☐ | ☐ |
| Were the participants included in any comparisons similar? | ☐ | ☐ | ☐ | ☐ | ☐ |
| Were the participants included in any comparisons receiving similar treatment/care, other than the exposure or intervention of interest? | ☐ | ☐ | ☐ | ☐ | ☐ |
| Was there a control group? | ☐ | ☐ | ☐ | ☐ | ☐ |
| Were there multiple measurements of the outcome both pre and post the intervention/exposure? | ☐ | ☐ | ☐ | ☐ | ☐ |
| Was follow up complete and if not, were differences between groups in terms of their follow up adequately described and analyzed? | ☐ | ☐ | ☐ | ☐ | ☐ |
| Were the outcomes of participants included in any comparisons measured in the same way? | ☐ | ☐ | ☐ | ☐ | ☐ |
| Were outcomes measured in a reliable way? | ☐ | ☐ | ☐ | ☐ | ☐ |
| Was appropriate statistical analysis used? | ☐ | ☐ | ☐ | ☐ | ☐ |
| N/A: not applicable | | | |  |  |

Critical appraisal tool for economic evaluations:

| Item | Yes | Partially | No | Unclear | N/A |
| --- | --- | --- | --- | --- | --- |
| Is there a well-defined question? | ☐ | ☐ | ☐ | ☐ | ☐ |
| Is there comprehensive description of alternatives? | ☐ | ☐ | ☐ | ☐ | ☐ |
| Are all important and relevant costs and outcomes for each alternative identified? | ☐ | ☐ | ☐ | ☐ | ☐ |
| Has clinical effectiveness been established? | ☐ | ☐ | ☐ | ☐ | ☐ |
| Are costs and outcomes measured accurately? | ☐ | ☐ | ☐ | ☐ | ☐ |
| Are costs and outcomes valued credibly? | ☐ | ☐ | ☐ | ☐ | ☐ |
| Are costs and outcomes adjusted for differential timing? | ☐ | ☐ | ☐ | ☐ | ☐ |
| Is there an incremental analysis of costs and consequences? | ☐ | ☐ | ☐ | ☐ | ☐ |
| Were sensitivity analyses conducted to investigate uncertainty in estimates of cost or consequences? | ☐ | ☐ | ☐ | ☐ | ☐ |
| Do study results include all issues of concern to users? | ☐ | ☐ | ☐ | ☐ | ☐ |
| Are the results generalizable to the setting of interest in the review? | ☐ | ☐ | ☐ | ☐ | ☐ |
| N/A: not applicable | | | |  |  |
