# Supplementary material for: Implementation strategies and economic considerations for point-of-care ultrasound in Low- and Middle-Income Countries: A scoping review
Source: PLOS Glob Public Health. 2026 Feb 9;6(2):e0005852. doi: 10.1371/journal.pgph.0005852 (PMC12885260; doi:10.1371/journal.pgph.0005852)
Supplement: S3 Text — (DOCX) [file pgph.0005852.s004.docx]

**S3 Text: Quality Assessment**

| *JBI Checklist for quasi-experimental studies* | | | | | | | | | | | | |
| --- | --- | --- | --- | --- | --- | --- | --- | --- | --- | --- | --- | --- |
| First author, year of publication | Clear research question | Participants | Intervention | Control Group | Multiple measurements | Follow-Up | Methods for outcome measurement | Outcome measurement | Statistical analysis |  |  | Overall quality assessment |
| Aeberlie, 2018 | ✓ | ✓ | ✓ |  |  | (✓) |  |  |  |  |  | Low |
| Aspler, 2022 | (✓) | ✓ | ✓ |  | (✓) | (✓) | (✓) | ✓ | (✓) |  |  | Moderate |
| Boamah, 2014 | ✓ | (✓) | (✓) |  | (✓) | ✓ | ✓ | ✓ | (✓) |  |  | Moderate |
| Dreyfuss, 2020 | ✓ | ✓ | ✓ |  |  | ✓ | ✓ | ✓ |  |  |  | Moderate |
| Hall, 2021 | ✓ | (✓) | ✓ |  | ✓ | ✓ | ✓ | ✓ | ✓ |  |  | High |
| Viner, 2022 | ✓ | ✓ | ✓ |  | ✓ | ✓ | ✓ | ✓ | ✓ |  |  | High |
|  | | | | | | | | | | | | |
| *JBI Checklist for Economic Evaluations* | | | | | | | | | | | | |
| First author, year of publication | Clear research question | Description of alternatives | Relevant costs and outcomes identified | Established clinical effectiveness established | Accurate costs and outcome measurements | Costs and outcomes valued credibly | Adjusted for differential timing | Incremental analysis | Sensitivity analysis | All issues of concerns included | Generalizability | Overall quality assessment |
| Bresnahan, 2021 | ✓ | ✓ | (✓) | (✓) | (✓) | ✓ | ✓ | ✓ | ✓ | ✓ | (✓) | High |
| Elzarek, 2014 | ✓ | ✓ | (✓) | ✓ | (✓) | ✓ |  | (✓) |  | (✓) | (✓) | Moderate |
| Kimura, 2024 | ✓ | ✓ | ✓ | ✓ | (✓) | ✓ |  |  |  | ✓ | (✓) | Moderate |
| Nambaziira, 2022 | ✓ | ✓ | (✓) | ✓ | ✓ | ✓ | (✓) | ✓ |  | ✓ | (✓) | High |
| Pigeolet, 2024 | ✓ | ✓ | ✓ | (✓) | (✓) | ✓ | ✓ | ✓ | ✓ | ✓ | (✓) | High |
| Ponde, 2016 | ✓ | ✓ | (✓) | (✓) | (✓) | (✓) |  | (✓) |  | (✓) | (✓) | Moderate |
| JBI: Joanna Briggs Institute, ✓= Yes (2 points), (✓) = Partially (1 point) | | | | | | | | |  |  |  |  |

The identified implementation studies had a clear research question. Qualification and POCUS experience of study participants varied, potentially influencing outcomes. The majority of studies employed a before-and-after design to assess skill acquisition and knowledge retention in their programme. Consequently, no study included a control group. Assessments of successful implementation were typically performed immediately following the intervention with short follow-up measurements carried out 3-6 months later. One study specifically addressed feasibility aspects of POCUS implementation regarding remote mentoring, but was rated as low quality due to inconsistent outcome measurement methods and inadequate follow-up procedures [34]. For economic evaluations, all studies stated clear research questions, but the identification and assessment of relevant costs and outcomes were only partially fulfilled. As most studies evaluated very specific interventions within a specific context, the generalisability of findings was limited. However, three studies accounted for standardisation of their calculations to make them comparable across different settings [40, 43, 44] and over time and two studies included a sensitivity analysis to assess the robustness of its findings [40, 44].
