# Supplementary material for: Implementation strategies and economic considerations for point-of-care ultrasound in Low- and Middle-Income Countries: A scoping review
Source: PLOS Glob Public Health. 2026 Feb 9;6(2):e0005852. doi: 10.1371/journal.pgph.0005852 (PMC12885260; doi:10.1371/journal.pgph.0005852)
Supplement: Attachment — Submitted filename: REVIEW_PGPH-D-25-02260.docx [file pgph.0005852.s005.docx]

MINOR REVISION:

- Authors should specify the year in which the World Bank classified these countries as LMICs, given that this is dynamic.

- Explain the cleaning procedure in Excel (line 157).

- Explain how the checklists were adapted (line 178). This will make it easier to understand.

- The results are essentially descriptive; consider linking the Implementation and Economy dimensions.

- Give more weight to your approach on the reverse use of CFIR-ERIC.

- Discuss the limitations of the CFIR-ERIC tool.

MAJOR REVISION:

- To ensure the quality of the study selection process, inter-rater reliability should have been determined;

- Line 180: is the scoring standard? If not, explain the reasons for this adaptation and how it was done.
