# Supplementary material for: Implementation strategies and economic considerations for point-of-care ultrasound in Low- and Middle-Income Countries: A scoping review
Source: PLOS Glob Public Health. 2026 Feb 9;6(2):e0005852. doi: 10.1371/journal.pgph.0005852 (PMC12885260; doi:10.1371/journal.pgph.0005852)
Supplement: Attachment — Submitted filename: PbP Response PGPH_final.docx [file pgph.0005852.s006.docx]

Dear Dr Naveed, dear Editors, dear Reviewers,

we thank you for the time and effort invested in reviewing our manuscript titled ‘Implementation strategies and Economic Considerations for Point-of-Care Ultrasound in Low-Resource Settings: A Scoping Review’.

We have carefully considered and addressed each of the reviewers' comments. In our response below, we have detailed each comment and highlighted our feedback in green for clarity. We have also included references to specific lines where the revisions have been implemented in the manuscript. Line numbering refers to the unmarked version of the manuscript.

We believe these changes have strengthened the manuscript and look forward to your consideration for publication. Thank you once again for your feedback.

We are looking forward to hearing from you.

Best regards

Dr Benno Kreuels

Reviewers' comments:

Reviewer #1: Regarding the manuscript titled: Implementation Strategies and Economic Considerations for Point-of-Care Ultrasound in Low-Resource Settings: A Scoping Review. The authors conducted a scoping review of 12 studies that implemented point-of-care ultrasound (POCUS) in low-resource settings (LRS). Six studies focused on implementation strategies, and six studies evaluated economic aspects. While the topic is important and relevant.
Response: We thank the reviewer for acknowledging the importance and relevance of our topic.

Reviewer #1: I have the following comments:
Methods:
All the included studies are either cross-sectional or prospective in design; no randomized trials were identified. This should be clearly stated in the limitations.
Response: We clarified this in the limitation section (page 24, line 421-423)

Reviewer #1: Introduction:
The opening sentence describing challenges of CT and MRI is redundant and does not add value to the introduction. I recommend removing it. Instead, the introduction should focus more directly on comparing the challenges of comprehensive ultrasound versus POCUS in low-resource settings.
Response: We have shortened the introductory section to lead more directly into the distinction between comprehensive ultrasound and POCUS as well as the challenges relevant to the settings described (page 4, line 68-71).

Reviewer #1: Results:
Please add patient numbers for each study in Tables 1 and 2.
Response: We included number of study participants in the implementation studies as suggested to better reflect small scale project (Table 1). Additionally, the population or investigations used for cost estimates were added to Table 3.

Reviewer #1: The results section is excessively long (up to 12 pages), which makes it difficult to follow. The authors should prioritize the most relevant findings that directly address the aim of the review. Less essential results could be moved to supplementary material. Consider avoiding duplication of findings: if results are presented in a table or a figure, there is no need to fully reproduce them in the text.
Response: We thank the reviewer for this comment. The quality assessment was condensed, with key findings now summarised in S2, in line with the comments of Reviewer 3 (page 11, lines 221–225; S2). Overall, the results section was shortened to prioritise key findings while avoiding repetition as suggested.

Reviewer #1: Discussion:
The discussion is poorly structured. The key findings should be summarized clearly at the beginning, but instead, two pages are spent re-describing results already presented in the Results section. The authors should review and compare their findings with results from other studies, particularly contrasting experiences in low- and high-income countries.
Response: We have substantially restructured the discussion section. Main findings were summarised concisely and repeated descriptions of results already presented before were removed. As we aimed to identify key strategies for success implementation, we integrated a more in-depth discussion of key aspects, including the comparison of findings with the Knowledge-to-Action process and clarification of our use of the CFIR–ERIC tool, in line with comments from Reviewers #2–4. We also added a paragraph comparing our findings with recent work by Eppel et al. (page 22, 386-390), and with a recently published long-term assessment of an implementation initiative from Kenya (page 21, 364-368). We chose not to compare our findings to programmes from high-income settings as long-term evaluations are similarly scarce and diverse in design and follow up. Given the substantial differences in resources and implementation contexts, we judged that direct comparisons would be of limited interpretability and therefore did not include them.

Reviewer #1: Formatting: The manuscript contains inconsistent use of fonts and font sizes. Please ensure a uniform font type and size throughout.
Response: We thank the reviewer for pointing out this formatting issue. We have corrected these and ensured consistent formatting upon resubmission.

Reviewer #2: The study aims to identify implementation strategies and highlight their challenges and approaches for integration along with the cost effectiveness analysis. The study presented by the authors have considered only 12 studies (6 implementation, 6 economic evaluations) which appears to be inadequate to draw scientific coherent conclusion. The evidence base is found to be thin, fragmented, and dominated by short-term, externally led projects. While the scoping review attempts to address the concerns of POCUS implementation strategies on ground but lacks strong evidence to policy recommendations. The study does not provide definitive guidance on cost-effectiveness or large-scale implementation of POCUS in LMICs. Only 12 studies were included. Many are small-scale, single-country, short-term interventions with weak designs (mostly observational, no control groups). This limits generalizability.
Response: We agree with the concerns raised and fully acknowledge that the available evidence base is limited, which restricts the generalisability of findings. We have amended the Discussion section also in accordance with the related comments of Reviewer 1 for clarification (page 23, line 409-417).
We would like to clarify that we did not aim to provide sufficient evidence for policy recommendations rather than mapping evidence on successful strategies that could be useful for further structured assessments. We were able to identify key aspects relevant for future research and implementation programmes, including well-planned, adaptable, and context-specific implementation strategies built on equal partnerships to enhance sustainability (page 22, line 384-390). We further emphasised the importance of embedding economic evaluations within such implementation programmes to enable evidence-based decision-making (page 23, line 402-412).
Unfortunately, no studies identified to date fulfilled all of these criteria. However, well conducted programmes acknowledging the above could provide better evidence and in the next step guide policy recommendations for the implementation of POCUS in LMICs.

Reviewer #2: While CFIR-ERIC tool strengthens interpretation, moving beyond description to actionable strategy recommendations, barriers were poorly reported in included studies, forcing the authors to use facilitators “in reverse.” This is innovative but methodologically debatable, as acknowledged by the authors. The studies on implementation strategies are mainly observational; hence, this does not give any recommendations on the implementation strategy to be adopted.
Response: We appreciate the reviewer’s comments and the acknowledgement of our efforts to advance the use of the CFIR–ERIC tool. As noted, we addressed the methodological limitations of applying the tool in a reverse manner in the original manuscript, and we have further emphasised these constraints in the revised version (page 22, line 373-383).
However, our findings showed substantial overlap between the outputs generated with and without the additional “reverse” facilitator input. Importantly, the key aspects identified as relevant for improving POCUS implementation remained consistent.

Reviewer #2: The study has considered limited data on the cost impact analysis from the health system/provider perspective. The study should have included direct & indirect costs involved in POCUS intervention as compared to the existing practice. The study has also not included the incremental cost utility ratio (ICUR) for a better understanding and assessment of the intervention. There is a need to incorporate equity, patient-level costs, and contextual adaptations into economic analyses.
Response: We thank the reviewer for this important aspect. We summarised the direct and indirect costs reported in the identified studies and presented them in the Results section (page 18, line 327-331). Individual cost items were also included in Table 3. None of the included studies explicitly reported incremental cost-utility or incremental cost-effectiveness ratios (ICUR, ICER), although three studies potentially provided parameters necessary for such calculations (Bresnahan et al., Nambaziira et al., Pigeolet et al.). However, given the heterogeneity of employed methodologies and reported cost components, we did not undertake a secondary analysis. We have emphasised the need for future research incorporating patient-focused cost-utility or cost-effectiveness analyses to enable more robust economic assessments (page 23, 402-409).

Reviewer #3: Scope and Objectives:
The objectives could be more clearly differentiated between implementation strategy mapping and economic evaluation synthesis. Currently, both are discussed together throughout the text, which can make it difficult to follow the distinct analytical processes.
Consider briefly stating in the Methods how these two review components were conceptually linked.
Response: We included a short clarification in the Methods section to underline our objectives of mapping evidence on POCUS implementation programmes, systematically assessing their challenges, and evaluating their economic implications (page 6, line 120-123).

Reviewer #3: Methodological Transparency:
The search end date is listed as April 30, 2025—please clarify if this is a projected cut-off or a typographical error
Response: The initial search included literature up to February 2024. To ensure the most up-to-date results, we extended the search to include additional studies published between March 2024 and April 2025, particularly given the limited number of studies identified in this field. This reflects an intentional update rather than a typographical error or projected cut-off.

Reviewer #3: Provide the full search strategy as a supplementary file (as referenced in OSF) to enhance reproducibility.
Response: The search strategy has been added as Supplement 1 for transparency as suggested.

Reviewer #3: Application of the CFIR–ERIC Tool:
The adaptation of the tool to infer facilitators is innovative but unconventional. Please elaborate on how this reverse application was validated or justified, with references to prior work.
Response: Several studies have previously explored using the CFIR-ERIC tool in reverse to advance its utility. Developers and users of the tool acknowledged its limited adoptability in practice due to complexity. In line with previous comments, we further clarified this approach in our Discussion (page 22, line 373-383).

Reviewer #3: Results:
The quality assessment results could be summarised in one paragraph rather than dispersed across sections.
Response: Thank you for this suggestion. We have summarised the overall quality assessment in a short paragraph and moved detailed information to S2, in accordance with the comments of Reviewer #1 (page 11, line 221-225).

Reviewer #3: Discussion:
The discussion could better integrate how identified implementation strategies align with or diverge from prior POCUS training reviews (e.g., Eppel et al., 2025).
Response: We have added a dedicated paragraph that integrates our findings with the mentioned POCUS training review. While Eppel et al. primarily examined challenges and design features of individual training programmes, our review focuses on broader implementation strategies applicable to larger-scale initiatives. Areas of alignment are described in the discussion section (page 22, line 386-390).

Reviewer #3: Minor Comments
1. Proofread for consistency in referencing style and punctuation (some references lack DOIs or journal issue numbers).
Response: We thank the reviewer for highlighting this important point. The references have been updated, and full information has been included where available.

Reviewer #3: 2. Standardize terminology (e.g., “low-resource settings,” “LMICs,” “resource-constrained settings”).
Response: We reviewed the manuscript and used LMIC throughout the manuscript to avoid further confusion.

Reviewer #3: 3. The abstract could be more quantitative—e.g., report the number of included studies per region and methodological quality category.
Response: As suggested, we have added the number of included studies per region to the abstract (page 2, line 40-42). Given the detailed nature of the quality assessment and the limited space available in the abstract, we believe this information is more appropriately presented in the main manuscript avoiding overloading the abstract with methodological detail.

Reviewer #3: 4. Ensure figure legends are self-contained and that figure quality meets journal standards.
Response: We thank the reviewer for this important point. We reviewed the quality of our figures as well as their legends to meet scientific publication requirements.

Reviewer #3: 5. Minor English language polishing would improve readability, particularly in the Results and Discussion sections.
Response: The results and discussion section were reviewed for scientific language and amended accordingly.

Reviewer #4: MINOR REVISION:
- Authors should specify the year in which the World Bank classified these countries as LMICs, given that this is dynamic.
Response: We used the latest World Bank definitions as of 2025 and added the clarification in the manuscript (page 6, line 130).

Reviewer #4: - Explain the cleaning procedure in Excel (line 157).
Response: Due to differences in wording and summarisation of findings between the data extractors, the extracted data were aligned in Microsoft Excel. We added a short statement for clarification (page 8, line 159-160).

Reviewer #4: - Explain how the checklists were adapted (line 178). This will make it easier to understand.
Response: We have clarified in the manuscript that we introduced a “partially fulfilled” option for each item to improve the reflection of study quality (page 9, line 182-184).

Reviewer #4: - The results are essentially descriptive; consider linking the Implementation and Economy dimensions.
Response: We appreciate this suggestion. However, none of the identified studies combined implementation strategies and economic analyses within the same programme or setting. We therefore chose to present results separately to avoid associations that the underlying data does not support.
In line with previous comments, we have strengthened the Discussion to emphasise the importance of conducting economic evaluations alongside implementation studies in future initiatives to provide a more comprehensive understanding of feasibility, sustainability, and resource implications.

Reviewer #4: - Give more weight to your approach on the reverse use of CFIR-ERIC.
Response: This has been addressed in line with previous comments (page 16, 284-392, page 22, line 373-383).

Reviewer #4: - Discuss the limitations of the CFIR-ERIC tool.
Response: This has been addressed in line with previous comments (page 22, line 373-376).

Reviewer #4: MAJOR REVISION:
- To ensure the quality of the study selection process, inter-rater reliability should have been determined;
Response: Unfortunately, we did not calculate formal inter-rater reliability statistics during the screening process. In line with the methodological guidance for scoping reviews by the JBI and PRISMA, two reviewers independently screened all titles/abstracts and full texts, followed by discussion of any discrepancies until consensus was reached. This consensus-based approach is widely applied in evidence syntheses and ensured a transparent and rigorous study selection process.

Reviewer #4: - Line 180: is the scoring standard? If not, explain the reasons for this adaptation and how it was done.
Response: Thank you for this comment. JBI does not recommend using its checklists to generate a formal numerical score per se as critical appraisal requires judgement and each item should be considered individually. To support this process, we introduced a “partially fulfilled” response option for items where criteria were only partly met, in order to better capture study quality. This adaptation facilitated discussion among authors when agreeing on the overall appraisal of each study, in line with JBI guidance. We have clarified this process in the manuscript (page 9, line 182-189).
